# Tracing the recent evolution of Group A *Streptococcus* M1 serotype

**DOI:** 10.1080/22221751.2026.2623691

**Published:** 2026-01-27

**Authors:** Olivia M. Bertolla, Mark J. Walker, Stephan Brouwer

**Affiliations:** Australian Infectious Diseases Research Centre and Institute for Molecular Bioscience, The University of Queensland, Brisbane, Australia

**Keywords:** Group A *Streptococcus*, M1 serotype, M1_UK_ lineage, molecular epidemiology, genomic evolution, pathogen surveillance

## Abstract

The twenty-first century has seen global surges in scarlet fever and invasive Group A *Streptococcus* (GAS) infections, partly driven by the emergence of the toxigenic M1_UK_ lineage. Characterized by increased SpeA superantigen expression and the stepwise accumulation of 27 single nucleotide polymorphisms (SNPs), M1_UK_ has become the dominant GAS *emm*1 lineage in Europe, Australia and Canada, representing a notable shift in GAS molecular epidemiology. Interestingly, other distinct *emm*1 variants have emerged in China and Denmark but are yet to expand globally in the same manner. This review examines the recent evolution of the GAS *emm*1 lineage, with emphasis on genomic and molecular drivers, highlighting the ongoing diversification of this pathogen and the need for continued surveillance and research.

## Introduction

Group A *Streptococcus* (GAS, *Streptococcus pyogenes*) is a Gram-positive, β-haemolytic, human-restricted bacterial pathogen of global health concern [1]. GAS is responsible for a wide range of disease manifestations including pharyngitis, impetigo, scarlet fever, streptococcal toxic shock syndrome (STSS), necrotizing fasciitis and septicaemia [2]. Moreover, repeated GAS infections can trigger autoimmune sequelae such as acute post-streptococcal glomerulonephritis, acute rheumatic fever and rheumatic heart disease [2]. It is estimated that GAS infection accounts for over half a million deaths worldwide annually, with a large component attributed to complications with rheumatic heart disease [1]. GAS has remained susceptible to β-lactam antibiotics, including penicillin, which is fortunate given the absence of a commercially available vaccine for protection against GAS infection. However, resistance to macrolide and tetracycline antibiotics is becoming more prevalent, and emerging reports of subclinical β-lactam non-susceptibility are a growing concern [3,4].

GAS is classified into over 250 *emm*-types based on the sequence of the hypervariable 5′ region of the *emm* gene, encoding the M protein, a key virulence factor. Epidemiologically, *emm*-types vary depending on geographical region and may display tissue tropism [5]. The *emm*1 type is a frequent cause of invasive and non-invasive infections in high income countries [5]. While the throat and skin are primary reservoirs for GAS, *emm*1 is predominantly recovered from throat-associated infections [5]. Herein, we focus on the recent evolution and epidemiology of the pandemic GAS *emm*1 type.

## M1_global_ emergence and dominance

**Figure 1.**
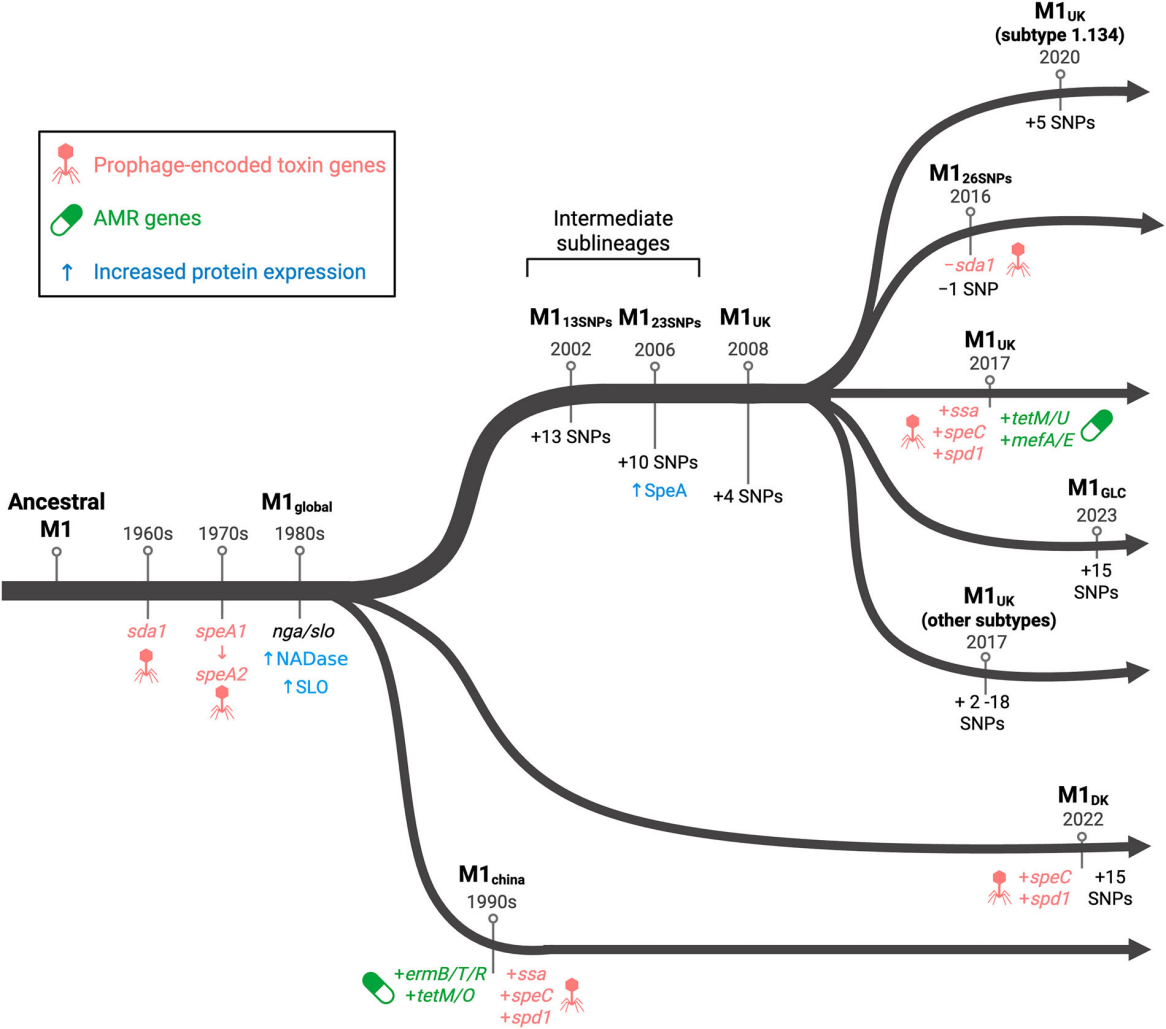
Molecular events contributing to GAS *emm*1 evolution. Genetic events that lead to the emergence of M1_global_ and subsequent M1 lineages. Ancestral M1 acquired two prophages harbouring *sda1* and *speA2* genes, followed by horizontal acquisition and recombination of the *nga*/*slo* locus from *emm*12 GAS resulting in increased expression of the *nga*/*slo* operon. The M1_UK_ lineage emerged following the stepwise accumulation of 27 SNPs and increased SpeA toxin expression. Genetic events associated with M1_UK_ sublineages and non-M1_UK_ lineages are indicated. Protein expression is indicated in blue. Phage encoded gene acquisition is indicated in orange. Antimicrobial resistance (AMR) gene acquisition is indicated in green. SNP, single nucleotide polymorphism. Created in BioRender. Walker, M. (2026) https://BioRender.com/cb5vr8u.

The early 1980s were marked by a sharp increase in invasive GAS infections in high-income settings – including Australia, the UK, Japan, Canada, and the USA – attributed to the emergence of a GAS *emm*1 clone designated M1T1, which gave rise to the M1_global_ clonal lineage [6–10]. The M1_global_ lineage differs from the less virulent ancestral M1 serotype in several aspects, including the stepwise acquisition of novel prophages encoding virulence factors deoxyribonuclease (DNase) Sda1 and the superantigen SpeA [11,12] (Figure 1). Subsequently, a single non-synonymous mutation converted *speA*1 to *speA*2, an allele that binds HLA-DQ with higher affinity and became selectively maintained in pandemic *emm*1 strains [13,14]. Following prophage acquisition, a horizontal transfer event with the GAS *emm*12 genotype led to the recombinational replacement of a 36-kb chromosomal region encoding the virulence factors Streptolysin O (SLO) and NAD-glycohydrolase (designated variously as NADase, SPN or NGA) [11,12]. Acquisition of two promoter SNPs and repair of a non-synonymous SNP in *nga* resulted in production of enzymatically active NADase and increased transcription of both toxin genes [12]. NADase and SLO are mutually interdependent for protein stability and synergistic for toxicity [12,15]. Enhanced virulence of M1_global_ was confirmed *in vivo*, with the M1_global_ strain MGAS2221 displaying increased virulence compared to the ancestral M1 strain SF370 in both murine and non-human primate models of necrotizing fasciitis and pharyngitis, respectively [11,12].

Scarlet fever is a toxin-mediated disease that was a significant cause of childhood morbidity in the 19th and early 20th centuries. Scarlet fever occurs when GAS strains that cause pharyngitis produce streptococcal pyrogenic exotoxins (superantigens) that act as potent activators of T-cells [16], leading to the characteristic systemic symptoms of the disease [2]. Over the last 200 years, global rates of scarlet fever steadily declined, and came to be regarded as a disease of the past. This decline was attributed to the introduction of antibiotics, improvements in hygiene, and increasing population immunity [17]. However, in 2011, a resurgence of scarlet fever was reported in mainland China and Hong Kong, associated with GAS *emm*12 and *emm*1 strains [18–21]. The majority of clinical isolates carried the superantigen-encoding genes *speC* and *ssa* and the DNase gene *spd*1, acquired via novel prophages circulating in this geographical region. SpeC and Spd1, but not SSA, have been shown to function synergistically to promote *emm*12 nasopharyngeal colonization in a murine model [22]. Additionally, horizontally acquired integrative and conjugative elements carrying antibiotic resistance genes for macrolide (*ermB/T/R*) and tetracycline (*tetO*/*M*) resistance were commonly detected in outbreak strains [21]. Together, these molecular events may have contributed to the evolution and dissemination of the pandemic M1_global_ lineage.

The incidence of *emm*1 GAS infections changed significantly during and after the COVID-19 pandemic. Similar to trends observed with *Streptococcus pneumoniae*, *Haemophilus influenzae*, and *Neisseria meningitidis*, a global decrease in reported GAS infections occurred in 2020 and 2021 [23–28]. This decline was likely driven by increased hygiene, social distancing, mask mandates, school closures, and other non-pharmaceutical interventions implemented during the pandemic. In England, a noticeable decline in GAS *emm*1 isolates from both adults and children was observed, likely linked to the respiratory transmission route and throat tropism of the M1 serotype [26]. However, following the relaxation of non-pharmaceutical interventions in 2022, this declining trend reversed dramatically with a global surge in GAS *emm*1 infections, with increases in tonsillopharyngitis cases, scarlet fever, and invasive GAS infections, including pneumonia and STSS [24–26,29–34]. In 2024, Japan reported its highest recorded number of STSS cases to date, prompting nationwide health concerns [35]. Reports of co-infection with respiratory viruses such as influenza, varicella, and COVID-19 were also associated with the rise in GAS infections [30,36–40]. Viral infections can disrupt mucosal barriers and modulate the innate immune system, predisposing individuals to secondary bacterial infections and potentially compounding disease severity [39,41]. These recent outbreak events have been associated, at least in part, with novel *emm*1 lineages such as the globally expanded lineage M1_UK_ [26] (Figure 1). In addition to M1_UK_, novel *emm*1 variants have emerged in China and Denmark, both associated with the aforementioned GAS outbreaks [18–21,29,42] (Figure 1).

## Emergence and dominance of the M1_UK_ lineage

**Figure 2.**
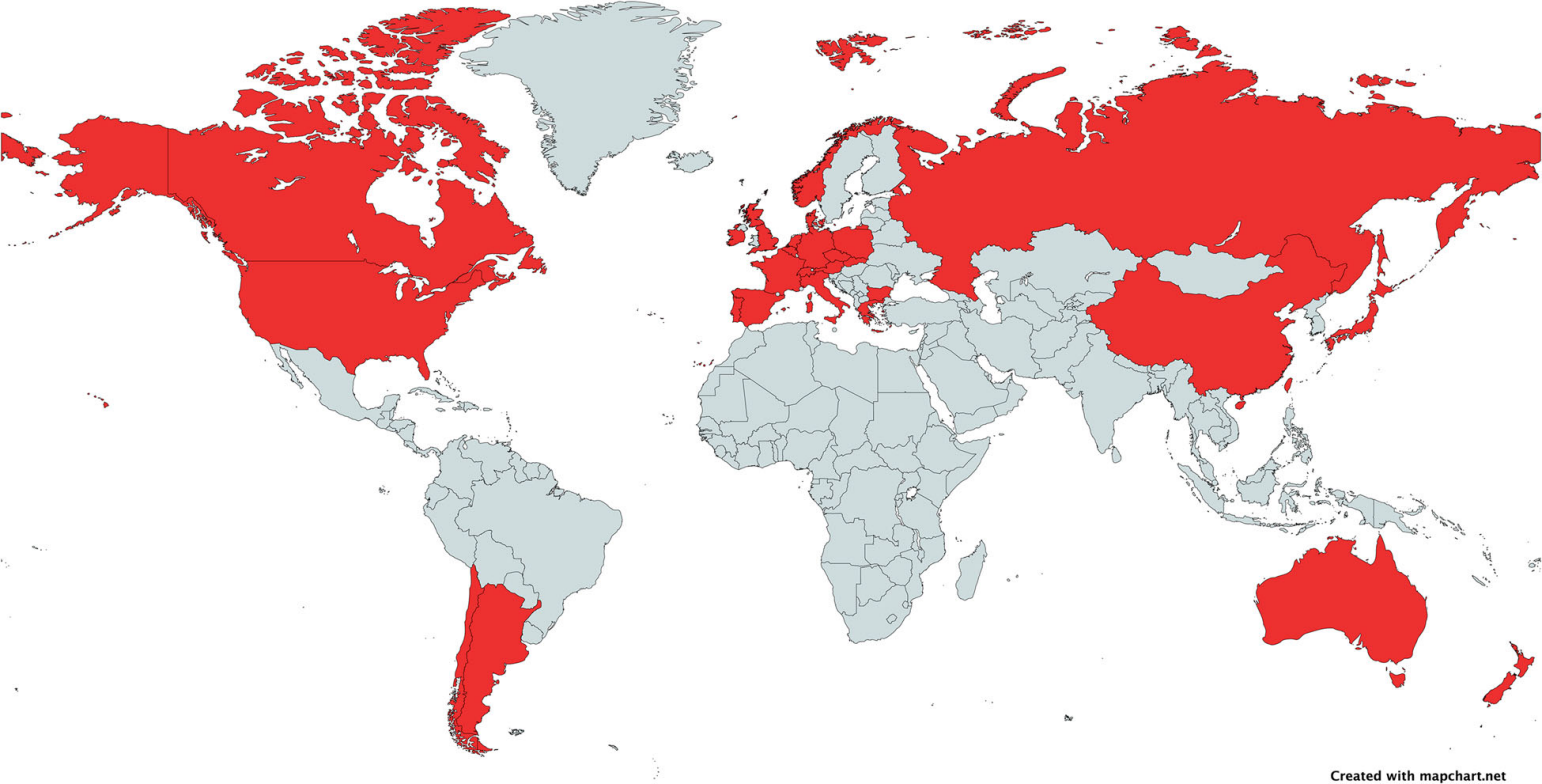
Global expansion of the GAS M1_UK_ lineage as of December 2025. Countries in red have reported M1_UK_ isolates. Data collected from PubMed using search terms “*Streptococcus pyogenes*”, “Group A *Streptococcus*” and “M1UK”. No language restrictions were applied. The timeframe spanned January 2019 to December 2025. Created with mapchart.net and reproduced under CC BY-SA 4.0 license (https://creativecommons.org/licenses/by-sa/4.0/).

An unprecedented resurgence of scarlet fever was reported in the UK between 2014 and 2016, associated with GAS *emm*3, *emm*12, *emm*1 and *emm*4 genotypes [43]. This increase in scarlet fever cases coincided with a parallel rise in invasive GAS infections, linked to the emergence of a new GAS *emm*1 lineage designated M1_UK_ [44]. Since its first detection in the UK in 2008, M1_UK_ has disseminated globally with detection in Europe [26,29,32,45–53], North America [31,54], South America [55], Asia [37,42,53,56] and Oceania [57,58] (Figure 2). Multiple studies have reported clonal replacement of M1_global_ by M1_UK_ within the GAS *emm*1 population. M1_UK_ is now the predominant *emm*1 lineage in several countries, including the UK, Belgium, Czech Republic, Australia, Japan, and Canada [26,31,49,52,57,59].

M1_UK_ has been reported as highly prevalent in specific clinical presentations, such as pneumonia, pleural empyema, and meningitis [26,60,61]. In the USA, anecdotal evidence has suggested a higher case fatality rate for M1_UK_ infections; however, such observations have not been statistically supported [60]. Similarly, studies from Spain, Belgium, Germany and Canada have reported no significant difference in clinical severity between M1_UK_ and M1_global_ infections [45,46,52,62]. Further clinical research is needed to fully understand the factors contributing to the dominance of M1_UK_ within the *emm*1 population.

Antibiotic resistance has been reported in M1_UK_ clinical isolates. In the original description of M1_UK_ by Lynskey and colleagues (2019), the *mefA* and *msrD* macrolide-resistance locus was identified in a single isolate [44]. However, in Belgium (2022–2023), both macrolide (*mefE*) and tetracycline (*tetU* and *tetM*) resistance genes were detected in M1_UK_, with higher carriage rates observed in M1_UK_ compared to M1_global_ [52]. Similarly, an M1_UK_ isolate from Greece exhibited resistance to erythromycin and clindamycin [51]. While antibiotic resistance levels in M1_UK_ remain relatively low, ongoing surveillance is warranted given its expanding prevalence and clinical significance.

Mutations in the genes encoding the two component regulatory system CovR/CovS (CovR/S) system are known to enhance the invasive potential of GAS isolates [63]. The CovR/S system consists of a transcriptional regulator (CovR) and a sensor kinase (CovS), together regulating up to 15% of GAS genomic content. Under environmental stresses such as elevated temperature (40 °C), high salt concentration, and iron limitation, the CovR/S regulatory system modulates the expression of multiple virulence-associated genes, including *slo* and *nga*, *spyCEP* (interleukin-8 protease), *sda*1 (DNase), and *hasABC* (hyaluronic acid capsule) [63–65]. Mutations in either *covR* or *covS* can lead to upregulation of these virulence-associated genes and repression of SpeB (cysteine protease) expression [63]. Although *covR/S* mutations are generally associated with a hypervirulent phenotype, they may impair the ability of M1_global_ to colonize and transmit to new hosts [66]. In comparison to M1_global_, M1_UK_ isolates appear to exhibit reduced genomic diversity with a reported lower frequency of *covR/S* mutations [26]. Hypothetically, this reduced mutation rate might confer a fitness advantage, suggesting that the 27 M1_UK_-defining SNPs could reduce the selective pressure for *covR/S* mutation. Nonetheless, mutations in either *covR* or *covS* have been reported in M1_UK_ isolates from Australia, UK, and Portugal [26,34,67,68].

### Stepwise accumulation of SNPs in M1_UK_

M1_UK_ is differentiated from its progenitor, M1_global_, by the presence of 27 chromosomal SNPs, and is characterized by increased expression of the superantigen SpeA [44,57] (Figure 1, Table 1). Two major intermediate sublineages, M1_13SNPs_ and M1_23SNPs_, have been identified to date, each carrying a subset of the 27 M1_UK_-specific SNPs [53,69]. However, neither sublineage has expanded within the *emm*1 population to the same extent as M1_UK_, suggesting that the full set of 27 SNPs is required to confer a fitness advantage during human infection and transmission. Of these 27 SNPs, functional consequences have been reported for six (Table 1). Among the first 13 SNPs, three occurred in *rofA*, a standalone transcriptional regulator. RofA was originally characterized in the GAS *emm*6 genotype as a positive regulator of the fibronectin binding protein gene *prtF* [70], and a negative regulator of *speA* expression [71]. However, isogenic mutation of *rofA* in representative M1_global_ and M1_UK_ strains showed no effect on SpeA expression levels [57]. Although some strain-specific effects have been suggested, no conclusive role has been attributed to the *rofA* SNPs in the M1_UK_ lineage [57,72].

**Table 1.** Presence of single nucleotide polymorphisms (SNPs) in M1_13SNPs,_ M1_23SNPs_, M1_UK_, M1_26SNPs_, M1_GLC_ and M1_DK_. Tick (✔) indicates SNP presence; cross (X) indicates absence.

Position^+^	Locus	Gene	Product	SNP^++^	AA^+++^	M1_13SNPs_ [57,69]	M1_23SNPs_ [57,69]	M1_UK_ [57,69]	M1_26SNPs_ [26,34]	M1_GLC_ [49]	M1_DK_ [31]	Phenotypic consequence
8402	Spy0006	*trcF*	Transcription-repair coupling factor	C>A	R1056S	X	X	X	X	✔	X	Unknown
46613	Spy0028	*–*	Autolysin	A>G	Y85H	X	X	X	X	✔	X	Unknown
47867	Spy0029	*purD*	Phosphoribosylamine–glycine ligase	C>T	I280I	X	X	X	X	✔	X	Unknown
50024	Spy0031	*purK*	Phosphoribosylaminoimidazole carboxylase ATPase subunit	A>G	I297V	X	X	X	X	X	✔	Unknown
53213	Intergenic	*–*	–	T>C	–	X	X	X	X	✔	X	Unknown
115646	Spy0106	*rofA*	Transcriptional regulator protein	C>T	D491N	✔	✔	✔	✔	✔	X	Strain-specific transcriptomic changes [57,72]
116162				A>C	F319V	✔	✔	✔	✔	✔	X	
116163				C>A	M318I	✔	✔	✔	✔	✔	X	
250832	Spy0243	*–*	ABC transporter-associated protein	T>C	N248N	X	✔	✔	✔	✔	X	Unknown
513254	Spy0525	*–*	Galactose-6-phosphate isomerase LacB subunit	G>T	A116S	✔	✔	✔	✔	✔	X	Unknown
528360	Intergenic	*asnS*	ATP-binding protein	A>T	–	X	✔	✔	✔	✔	X	Unknown
563631	Spy0566	*sagE*	Streptolysin S putative self-immunity protein	G>A	A52T	X	✔	✔	✔	✔	X	Unknown
600192	Spy0596	* *	Arginine-binding protein	T>C	V228A	X	X	X	X	X	✔	Unknown
613633	Spy0609	*gacH*	Phosphoglycerol transferase	T>C	L310P	✔	✔	✔	✔	✔	X	Unknown
626494	Spy0623	*–*	Methyltransferase	G>A	L35L	✔	✔	✔	X^#^	✔	X	Unknown
655648	Intergenic	*–*	–	C>T	–	X	X	X	X	X	✔	Unknown
661707	Spy0656	*trmD*	tRNA (guanine-N(1)-)-methyltransferase	G>A	A44T	X	✔	✔	✔	✔	X	Unknown
711808	Spy0709	*pyrC*	Dihydroorotase protein	A>C	N170T	X	X	X	X	✔	X	Unknown
727057	Spy0723	*hflX*	GTP-binding protein	C>T	T271T	X	X	X	X	✔	X	Unknown
730823	Spy0727	*recJ*	Single-stranded-DNA-specific exonuclease	C>T	A336V	✔	✔	✔	✔	✔	X	Unknown
741956	Spy0739	*–*	Tetratricopeptide repeat family protein	G>A	Q144Q	X	X	X	X	✔	X	Unknown
751431	Spy0750	* *	ABC transporter ATP-binding protein	G>A	G148R	X	X	X	X	X	✔	Unknown
760241	Spy0757	*hylA*	Hyaluronate lyase precursor	G>A	T581I	X	X	X	X	✔	X	Unknown
784467	Spy0779	*–*	Putative membrane spanning protein	T>C	V147V	X	✔	✔	✔	✔	X	Unknown
798142	Spy0793	*pepV*	Dipeptidase PepV	C>T	I112I	X	X	X	X	X	✔	Unknown
819098	Spy0825	*murB*	UDP-N-acetylenolpyruvoylglucosamine reductase	G>A	A82T	X	✔	✔	✔	✔	X	Unknown
860387	Spy0870	*–*	ABC transport system ATP-binding/permease protein	G>A	E460E	X	X	X	X	✔	X	Unknown
874083	Spy0883	*–*	Ribonuclease HII	G>A	T243T	X	X	X	X	X	✔	Unknown
890776	Spy0903	*oadB*	Oxaloacetate decarboxylase beta chain	G>A	M19I	X	X	X	X	X	✔	Unknown
908687	Spy0920	*–*	UDP-N-acetylmuramoylpentapeptide-lysine N(6)-seryltransferase	C>T	E15*	X	X	X	X	X	✔	Unknown
923079	Spy0933	*–*	Putative NADH-dependent flavin oxidoreductase	G>A	A101V	X	✔	✔	✔	✔	X	Unknown
942633	Spy0951	*pstB*	Phosphate transport ATP-binding protein	G>A	H123N	X	X	✔	✔	✔	X	Unknown
948344	Spy0957	*–*	Myo-inositol-1(or 4)-monophosphatase	A>C	L139L	X	X	X	X	X	✔	Unknown
967436	Spy0980	*–*	Cation diffusion facilitator family transporter	A>G	K303R	X	X	X	X	X	✔	Unknown
983438	Intergenic	*–*	Transcriptional leader of *ssrA* (transfer-messenger RNA)	G>C	–	X	✔	✔	✔	✔	X	Increased *speA* expression [57]
1045871	Spy1073	*dltA*	D-alanine-activating enzyme	G>A	H440Y	X	X	X	X	X	✔	Unknown
1058475	Spy1084	* *	Outer surface protein	G>A	L133L	X	X	X	X	X	✔	Unknown
1062670	Spy1088	*obg*	GTP-binding protein OBG family	G>A	R26C	X	X	X	X	X	✔	Unknown
1066980	Spy1094	*–*	MFS superfamily transporter protein	C>T	G46D	X	X	X	X	✔	X	Unknown
1082253	Spy1108	*metK2*	S-adenosylmethionine synthetase	C>T	A221T	✔	✔	✔	✔	✔	X	Unknown
1085850	Intergenic	*–*	–	C>T	–	X	X	X	X	✔	X	Unknown
1122435	Spy1146	*holA*	DNA polymerase III subunit delta	C>T	D215N	X	X	X	X	✔	X	Unknown
1238124	Spy1282	*msrA*	Peptide methionine sulfoxide reductase	G>A	A32V	✔	✔	✔	✔	✔	X	Unknown
1238673	Spy1283	*tlpA*	Thiol:disulfide interchange protein	G>A	A71V	✔	✔	✔	✔	✔	X	Unknown
1251193	Spy1293	*–*	Hypothetical protein	G>A	S135L	X	X	✔	✔	✔	X	Unknown
1272188	Spy1313	*–*	Beta-glucosidase	T>C	E87G	X	X	X	X	X	✔	Unknown
1373176	Spy1400	*–*	PTS system, galactose-specific IIB component	C>A	M66I	X	X	✔	✔	✔	X	Unknown
1407497	Spy1439	*–*	Portal protein	C>T	G290E	✔	✔	✔	X^#^	✔	X	Unknown
1446116	Spy1490	*fabG*	3-oxoacyl-[acyl-carrier protein] reductase	C>T	T231T	✔	✔	✔	✔	✔	X	Unknown
1484362	Spy1529	*shp*	Heme-binding Shp domain-containing protein	C>A	S97S	X	X	X	X	X	✔	Unknown
1514801	Intergenic	*–*	–	C>T	–	X	X	X	X	✔	X	Unknown
1535209	Intergenic	*–*	–	A>G	–	X	X	✔	✔	✔	X	Unknown
1681178	Spy1718	*sic*	Streptococcal inhibitor of complement protein	T>A	Q257L	X	X	X	X	✔	X	Unknown
1702540	Spy1741	*gldA*	Glycerol dehydrogenase	C>T	W175*	X	✔	✔	✔	✔	X	Premature stop codon, loss of GldA activity [69]
1734573	Spy1772	*ftcD*	Glutamate formiminotransferase	C>T	A2V	X	X	X	X	✔	X	Unknown
1734749				G>A	A61T	✔	✔	✔	✔	✔	X	Unknown
1828734	Spy1860	*–*	Putative membrane spanning protein	G>A	G71R	X	✔	✔	✔	✔	X	Unknown

^+^ Reference genome MGAS5005 (GenBank CP000017.2). ^++^SNP, nucleotide change; ^+++^AA, amino acid change. * indicates a stop codon. ^#^ M1_26SNPs_ has a loss of a SNP in either locus Spy0623 or Spy1439.

A single SNP occurred in the transcriptional leader sequence of *ssrA,* a transfer-messenger RNA gene located approximately 1 kb upstream of *speA*. Repair of this *ssrA* SNP in M1_UK_ resulted in decreased *speA* expression, while introduction of the SNP into M1_global_ led to increased *speA* expression, comparable to M1_UK_ levels. The underlying molecular mechanism has been described, and it was found that this SNP increases complementarity between the *ssrA* leader sequence and its transcriptional terminator, leading to enhanced transcriptional read-through and, consequently, increased *speA* expression [57]. Elevated SpeA expression is observed in both M1_UK_ and the intermediate M1_23SNPs_ sublineage, which also carries the *ssrA* SNP [69]. This indicates that increased SpeA expression plays a role in M1_UK_ evolution, but the additional four SNPs unique to M1_UK_ are likely to contribute further to its overall fitness and epidemiological success.

A single SNP among the 27 M1_UK_-defining mutations occurred in *gldA*, the gene encoding glycerol dehydrogenase. GldA is a key enzyme in the streptococcal glycerol dehydrogenation pathway, catalysing the reversible conversion of glycerol to dihydroxyacetone (DHA) [73]. Both M1_UK_ and M1_23SNPs_ carry the SNP in *gldA* that introduces a premature stop codon, resulting in protein truncation and loss of enzymatic activity. Deletion of *gldA* in M1_global_ strains produces a phenotype comparable to M1_UK_ [69]. Interestingly, the glycerol dehydrogenation pathway has been considered essential in some bacteria, as the alternate glycerol metabolism pathway produces hydrogen peroxide as a toxic byproduct [73]. RNA sequencing of a *gldA* deletion mutant in M1_global_ revealed upregulation of adjacent operon genes, including *pflD, mipB*, and genes of the PTS cellobiose-specific IIC system. These genes are involved in carbohydrate metabolism, including the processing of fructose, DHA, and pyruvate, suggesting that shifts in metabolic activity may confer a fitness advantage to M1_UK_ [69]. The broader metabolic consequences of the full set of 27 SNPs in M1_UK_ remain to be elucidated.

The specific contributions of the final four SNPs to M1_UK_ fitness remain unknown. It appears that only two genes are commonly differentially regulated in M1_UK_ compared to M1_global_: *speA* and *glA* (also referred to as *glpF.2*) [57]. The *glA* gene encodes a putative glycerol aquaporin and is downregulated in M1_UK_, likely due to a single SNP located in its promoter region. Aquaporins are integral membrane proteins that facilitate the transport of water and small solutes, including glycerol, across the cell membrane [74]. Prokaryotic aquaporins have been characterized in a variety of species, including *Escherichia coli*, *Lactobacillus plantarum, Pseudomonas aeruginosa*, *Streptococcus oligofermentans, Streptococcus pneumoniae* and *Streptococcus suis* [69,75–77]*.* In addition to water and glycerol, some bacterial aquaporins have been shown to transport other small molecules such as hydrogen peroxide, urea, oxygen, and DHA [69,75–77]. Bioinformatic analysis indicates that GAS *glA* is similar to the *glpF3* family of aquaporins in *L. plantarum*, which are associated with water, glycerol, and DHA transport [69]. In *S. suis*, the aquaporin Aagp facilitates hydrogen peroxide transport and contributes to virulence in a murine infection model [76,77]. Furthermore, *S. suis glpF* and other glycerol metabolism-related genes are downregulated under oxidative stress conditions [76]. Oxidative stress is a key environmental stressor encountered by GAS during infection, particularly in the presence of host phagocytic cells such as neutrophils, which generate reactive oxygen species to promote bacterial clearance. The role of aquaporins in GAS pathogenesis, however, remains to be elucidated.

The remaining three SNPs occurred in genes with less well-characterized functions: *pstB,* which encodes a phosphate transport ATP-binding protein; Spy1293, which encodes a hypothetical protein; and Spy1400, the gene encoding the galactose-specific IIB component of the PTS system. To date, no phenotypic characterization has been reported regarding the impact of these SNPs on M1_UK_ evolution.

### Ongoing diversification of M1_UK_

As exemplified by the evolution of M1_global_, the acquisition of mobile genetic elements has played an important role in the ongoing evolution of *emm*1 GAS. Notably, 26% of Australian M1_UK_ isolates harboured a novel prophage, ΦSP1380.vir, encoding the superantigens SSA and SpeC, as well as the DNase Spd1 [57]. This same toxin combination was overrepresented in Asian *emm*1 and *emm*12 isolates associated with the 2011 scarlet fever outbreak, linked to the prophage ΦHKU488.vir [21]. ΦSP1380.vir shares 95% sequence identity with ΦHKU488.vir, which has also been detected in some Australian M1_global_ strains [57]. In contrast to the 26% prevalence of Australian M1_UK_ isolates carrying ΦSP1380.vir, <1% of M1_UK_ isolates in the UK carried the ΦSP1380.vir prophage [26]. Instead, 9% of M1_UK_ isolates from the region, carried prophage ΦSF370.1, which only carries *speC* and *spd1*. M1_UK_ isolates harbouring toxin-carrying prophage have since also been detected in Canada, USA, UK, Netherlands, and Taiwan [26,31,56,60,78]. The functional contribution of SSA, SpeC, and Spd1 to M1_UK_ colonization, transmission, or virulence has not yet been characterized. However, the horizontal transfer of prophage-encoded virulence factors between GAS lineages may further enhance the virulence potential of M1_UK_, posing a significant public health concern.

Additional to acquisition, prophage loss has also been reported in some M1_UK_ isolates. An M1_26SNPs_ sublineage of M1_UK_ has been reported with either a loss of the Φ5005.3 prophage, encoding *sda1* and carrying a non-synonymous M1_UK_-defining SNP, or reversion of a synonymous SNP in Spy0623 encoding a methyltransferase [26,34] (Figure 1). Additionally, multiple subclades have been noted in the UK and Netherlands, defined by the presence of up to 18 additional clade specific SNPs (Figure 1). Three new clades emerged in the UK during the 2022 upsurge, undergoing rapid nationwide expansion [26]. In the Netherlands, four M1_UK_ clades have emerged, including a subtype *emm*1.134 variant with an additional five SNPs, including a non-synonymous mutation in the *emm*1 gene [78].

In Iceland, GAS *emm*1 isolates collected in 2022–23 that were initially classified as M1_UK_ were found to carry an additional 15 SNPs in their core genome, forming a distinct M1_UK_ sublineage designated M1_GLC_ [50] (Figure 1, Table 1). Increases in invasive GAS infections during this period have been attributed to the emergence of M1_GLC_ [79]. Among 75 invasive and non-invasive isolates sampled between 2022 and 2023, *emm*1 was the dominant genotype, with 96% of *emm*1 isolates belonging to the M1_GLC_ sublineage, underscoring its rapid expansion and dominance in the Icelandic population [79]. Phylogenetic and epidemiological analyses suggest that M1_GLC_ may have originated in Scotland, and its introduction to Iceland was followed by rapid local dissemination [50]. Notably, four of the defining SNPs of M1_GLC_ (located in the genes *purD*, *pyrC*, *hflX*, and *sic*) have also been found in an M1_UK_ clade in the UK, clustering phylogenetically with the Icelandic isolates [26]. Although the phenotypic consequences of the additional 15 SNPs remain to be characterized, it is interesting to note that one of these SNPs occurred in *sic* (streptococcal inhibitor of complement), encoding a highly polymorphic protein known to disrupt human innate immunity and protect against antimicrobial peptides [80]. SIC has also been shown to enhance GAS *emm1* survival within murine macrophages and promote virulence in systemic infection models. Surprisingly, M1_GLC_ has not yet been reported outside of Iceland.

## Other *emm*1 lineages

### Emergence of M1_china_

A recent analysis of GAS isolates in China from 1993–2020 revealed that *emm*12 and *emm*1 were the dominant *emm*-types across five scarlet fever incidence peaks [42]. *Emm*1 remained the second most frequent scarlet fever-associated genotype in China, behind *emm*12. A dominant *emm*1 clade, designated M1_china_, has accounted for over 98% of clinical cases since the 1990s. M1_china_ shares a common ancestor with M1_global_ and likely emerged shortly after the expansion of M1_global_ (Figure 1). M1_china_ strains carry multidrug-resistant ICE elements conferring macrolide and tetracycline resistance along with prophage encoding *ssa*, *speC*, and *spd1* [42]. Interestingly, despite its global spread, the M1_UK_ lineage has not become established in mainland China; only a single M1_UK_ isolate was reported from a patient with scarlet fever in 2018 [42]. The reasons for this remain unknown.

### Emergence of M1_DK_

Prior to the implementation of COVID-19 related restrictions, M1_UK_ was the dominant cause of invasive GAS infections in Denmark; however, following the easing of social distancing restrictions, a post-pandemic surge in invasive GAS coincided with the emergence of a novel lineage designated M1_DK_ [29]. First identified in 2022, by 2023 M1_DK_ accounted for 30% of sequenced invasive GAS isolates, supplanting M1_UK_ as the predominant *emm*1 lineage in Denmark [29]. M1_DK_ lacks the 27 SNPs that define M1_UK_ and does not show increased SpeA expression. Instead, it is characterized by 15 unique SNPs and the presence of a prophage encoding SpeC and Spd1 [29] (Figure 1). M1_DK_ was overrepresented among invasive disease cases compared with non-*emm1* genotypes; however, infection with M1_DK_ was not associated with higher mortality or increased risk of intensive care admission compared with other *emm*1 or non-*emm*1 lineages [29]. Moreover, M1_DK_-like precursor strains have been detected in the Netherlands, where several M1_global_ isolates from 2018–19 carried 13 or 14 of the 15 M1_DK_-defining SNPs, with some also harbouring the toxin-carrying prophage [78]. Nonetheless, M1_DK_ did not further expand in the Netherlands. Additionally, approximately 1% of GAS *emm*1 isolates from Argentina, isolated during 2023, were identified as M1_DK_ [55]. To date, M1_DK_ has not been reported outside of these countries [47,52]. Among the 15 lineage-defining M1_DK_ SNPs, one introduces a stop codon in Spy0957, an aminoacyltransferease involved in peptidoglycan synthesis. Several non-synonymous SNPs map to genes involved in metabolic pathways. However, the contributions of these 15 SNPs to M1_DK_ fitness and virulence remain to be determined.

## Concluding remarks

GAS infections remain a significant public health concern, particularly in light of the marked post-pandemic upsurge in case numbers. The emergence, global spread and ongoing diversification of M1_UK_ and other *emm*1 lineages underscores the critical importance of continued genomic surveillance and routine sequencing of both invasive and non-invasive GAS isolates to monitor population shifts. A deeper understanding of the molecular mechanisms underlying pathogen virulence and transmission, such as the contribution of the 27 lineage-defining SNPs to M1_UK_ fitness, is essential to inform public health strategies. The development of an effective GAS vaccine remains a global health priority, as recognized by the World Health Organisation [81]. However, vaccine development is complicated by factors including *emm*-type diversity, antigenic variation, and concerns regarding potential autoimmune responses. Understanding the dynamic evolution of the GAS population is therefore crucial for designing vaccines with broad and lasting coverage. Future research should continue to investigate phenotypic variation and host–pathogen interactions, particularly those contributing to the fitness and transmission success of emerging lineages such as M1_UK_.
